# Study on the Up-Conversion Luminescence and Conductivity Behavior of p-Type NiO:Yb,Er Thin Films

**DOI:** 10.3390/ma16134637

**Published:** 2023-06-27

**Authors:** Haoming Wei, Yangqing Wu

**Affiliations:** School of Physics and Physical Engineering, Qufu Normal University, Qufu 273165, China; yqwu@qfnu.edu.cn

**Keywords:** NiO thin films, up-conversion, Li^+^ ions, high transparency

## Abstract

In this work, Li^+^-doped NiO:Yb,Er thin films are obtained via pulsed laser deposition. It was found that the films exhibit high transparency in the visible region and clearly red up-conversion luminescence under 980 nm excitation. Doping with Li^+^ can adjust the up-conversion emission intensity of the films. Moreover, all the films have p-type conductivity with a single activation energy of around 360 meV. The sheet resistivity of the films can be improved through changing the doping concentration of Li^+^ ions. Taken together, 5% is the best doping concentration for the potential application of this kind of film.

## 1. Introduction

Up-conversion (UC) luminescence is an anti-Stokes process that could convert low-energy photons to higher-energy photons. Recent years have seen a surge in interest in rare-earth semiconductors exhibiting UC luminescence due to their numerous uses in photonics, biological imaging, and photovoltaics [1,2,3,4,5]. The most efficient UC luminescence emission is based on Er ions in combination with Yb ions as sensitizers, which exhibits large anti-Stokes shifts, long up-conversion luminescence lifetimes, and superior photostability [6,7,8]. Fluorides, such as NaYF_4_, NaGdF_4_, and NaLuF_4_, are frequently utilized as the host matrix for the study of UC luminescence due to their high chemical stability and low phonon energy [9,10,11,12,13]. These efforts have greatly advanced the understanding of the UC luminescence mechanism and related applications. For example, Xu et al. synthesized NaYF_4_:Yb,Er particles and doped them into the hole transport layer (HTL) of perovskite solar cells (PSCs) [14]. Cho et al. designed nanostructured Ag backplanes with NaYF_4_:Yb,Er particles to enhance the performance of PSCs [15]. Recently, we increased the photocurrent of PSCs through adding UC nanoparticles to their absorption layer [16]. However, further enhancement of the performance of these photovoltaics is limited due to the fact that host materials of UC are highly insulated.

Since the photoelectric characteristics of oxide semiconductors may be easily regulated via ion doping, the realization of an effective UC luminescence based on oxide semiconductors has attracted significant attention. Several efforts have been made to dope rare-earth ions into TiO_2_ hosts, a well-known n-type semiconductor. Zhang et al. synthesized Yb/Er co-doped TiO_2_ nanocrystals and enhanced UC red light emission through introducing a pyrochlore phase [17]. Jung et al. prepared Er/Yb-doped TiO_2_ UC phosphors and found that UC emission might be improved through regulating the growth temperature [18]. Dong et al. realized multicolor tunable UC luminescence using a lower power modulation in the film of TiO_2_:Yb,Er [19]. In our previous work, we used pulsed laser deposition (PLD) to obtain TiO_2_:Yb,Er thin films and investigated the enhancing mechanism of the UC process with different doping concentrations of Mn^2+^ ions [20]. Moreover, much work has been expended to improve the photoelectric performance of PSCs through introducing TiO_2_-based UC materials [21,22,23,24]. These works demonstrate the feasibility of using doped-TiO_2_ with UC emission as an electrode for electron transport in PSCs. However, there has not been much research on UC emission in p-type oxides, which can be used as the HTL for PSCs.

NiO is a typical p-type semiconductor that has high work function and a suitable band gap. Inverted PSCs using NiO as the HTL exhibit a high open-voltage and stable power efficiency [25]. Although stoichiometric NiO is an insulator at room temperature, the conductivity of NiO can be increased via doping with monovalent elements, such as Cu and Li [25,26]. Hence, it is of clear significance to realize UC emission in NiO-based materials and control the transparency and conductivity for application as an HTL in PSCs. PLD is the most suitable growth method for oxide thin films with less impurities and better crystallinity. In this work, we grew various NiO thin films via PLD. All the films with Yb and Er doping exhibit clear UC emission in the visible range with the excitation of a 980 nm laser. Furthermore, the resistivity of NiO:Yb,Er films is significantly reduced when doping with Li^+^ ions. NiO:Yb,Er thin film with 5% Li^+^ doping concentration shows excellent transparency, electrical conductivity, and up-conversion luminescence, which can be used to extend the absorption spectra of PSCs to the infrared region.

## 2. Materials and Methods

Thin films were grown on single-crystalline Al_2_O_3_ substrate using PLD with a KrF laser of 248 nm wavelength. One stoichiometric NiO target and five doped NiO targets with different doping concentrations were prepared for deposition. In the doped targets, the concentrations of Yb and Er were fixed at 10% and 2%, and the Li^+^ concentrations were between 0% to 10%, as shown in Table 1. The distance between the substrate and PLD targets was set to 5.5 cm. The energy and repetition rate of the laser were set to 200 mJ and 5 Hz, respectively. The deposition temperature was 500 °C and the oxygen pressure was 1 mTorr for all the films during growth. After deposition, the samples were annealed in situ for 30 min at a temperature of 500 °C. The thickness of the films are around 20 nm, which was controlled via the number of laser pulses and measured using a Bruker Dektak-XT profilometer. The obtained thin films and related information are summarized in Table 1 and labeled as S0–S5.

The crystalline structure of the thin films was tested with wide-angle X-ray diffraction (WARD) using a RIGAKU’s SmartLab diffractometer (Cu-K_α_ radiation), which was equipped with a Bragg-Brentano goniometer. The morphology and chemical elements of films were studied via field emission scanning electron microscope (FE-SEM, Sigma 500 VP, Carl Zeiss AG, Jena, Germany) with energy-dispersive X-ray spectroscopy (EDX). An ultraviolet-visible absorption spectrometer was used to record the transmittance spectra (Lambda 1050, PerkinElmer, Waltham, MA, USA). The Al_2_O_3_ substrate has a transmittance above 90% in the visible range. When measuring the transmittance spectra of the films, the Al_2_O_3_ substrate was fixed on the path of the parallel light as reference. Hence, the obtained curves are the transmittance spectra of the films after substrate contribution was subtracted. The PL spectrum UC luminescence spectra were collected using a fluorescence spectrophotometer (FLS1000, Edinburgh, UK) equipped with a PMT-900 detector. The excitation source is a 980 nm laser which can adjust the output power from 0 to 1.4 W. The PL spectra of all the thin films were detected with an output power of 1 W. The in-plane resistivity and carrier concentration were examined using a Hall setup in van der Pauw geometry (8404, LakeShore, Carson, CA, USA).

## 3. Results and Discussion

### 3.1. Crystalline Structure and Surface Morphology

**Figure 1:**
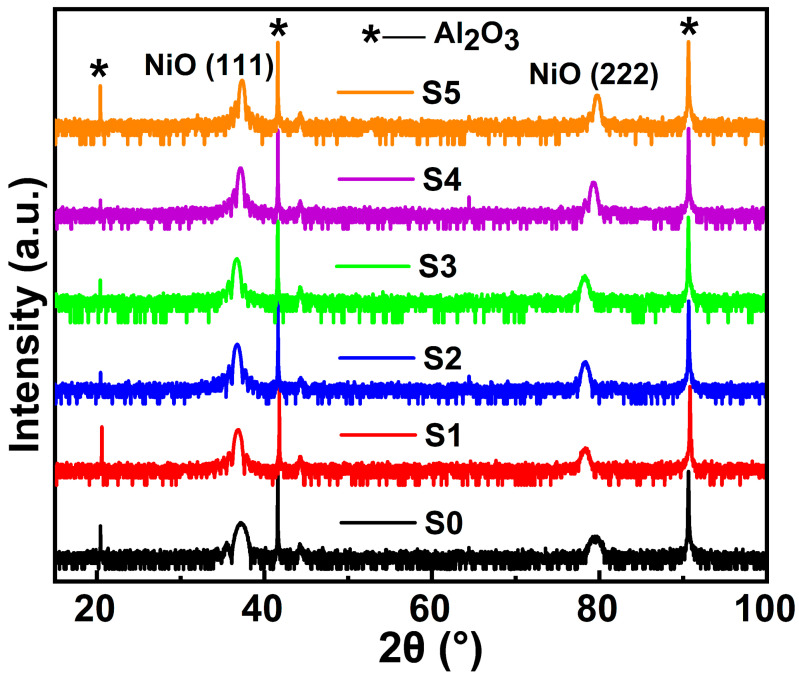
XRD patterns of pure NiO and Li^+^-doped NiO:Yb,Er thin films.

Since the films were deposited via PLD, except the elements in the target, no other elemental impurity can be introduced during the PLD process. The crystalline phase and the growth orientation of the obtained thin films were checked with WAXD 2*θ*-*ω* scans, as shown in Figure 1. Besides the diffraction peak with marks from the Al_2_O_3_ substrate, all other XRD peaks correspond to bunsenite NiO (JCPD card no. 47-1049), indicating that all the obtained samples have a single phase without any crystalline phase impurity. The XRD scans were detected in the Bragg–Brentano geometry; therefore, only the X-rays from the crystallographic planes that are parallel to the substrate can be detected. So, the strong (111) and (222) peaks indicated that all the films grown on the Al_2_O_3_ substrate are epitaxial with a certain out-of-plane orientation. The samples S4 and S5 have a high doping concentration, and the crystal volume may be reduced when more Ni ions are replaced by Li ions. Hence, the out-of-plane lattice constant decreases and results in a slight shift of XRD peaks. Furthermore, the grain size can be calculated based on the Debye–Scherrer formula. For the epitaxial thin films, only the grain size in the out-of-plane direction can be obtained because the films have a certain growth direction, as proved by 2*θ*-*ω* scans. The calculated out-of-plane grain size is around 10 nm, which slightly increases with doping of Li ions.

**Figure 2:**
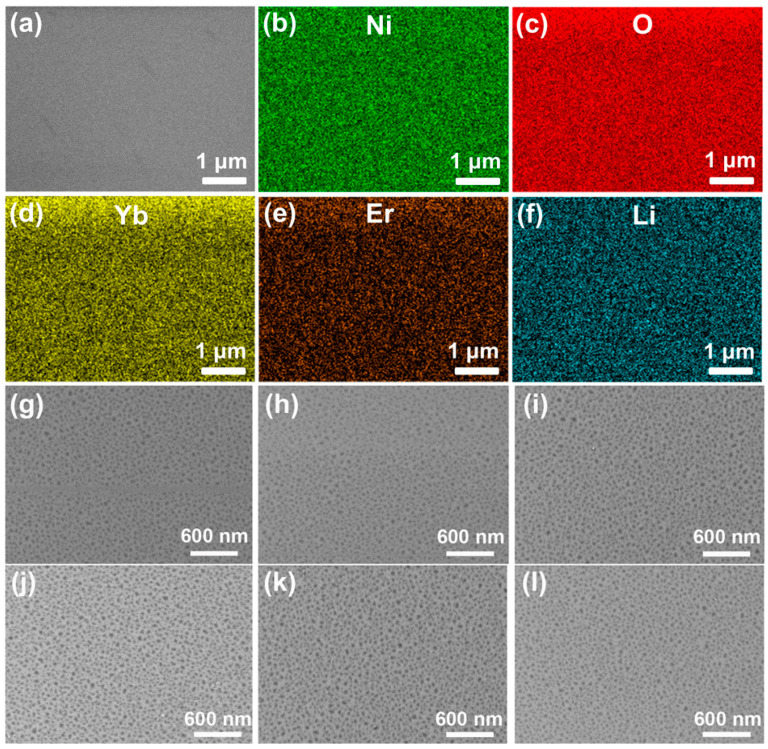
(**a**) SEM image of Li-doped NiO:Yb,Er thin film (S4). (**b**–**f**) EDX mapping of Ni, O, Yb, Er, and Li elements, respectively. (**g**–**l**) SEM image of S0–S5 at higher magnification.

Figure 2a shows the typical SEM image of Li^+^-doped NiO:Yb,Er thin film (S4). It confirmed that a flat and compact film is obtained. Chemical analysis of the Li^+^-doped NiO:Yb,Er film was checked through measuring EDS spectra of the sample. The characteristic X-ray of the selected elements was detected as displayed in Figure 2b–f. The EDS element mapping images revealed that Ni, O, Yb, Er, and Li existed in the film. However, the accurate analysis of Li-doped samples via EDS and other energy spectra is difficult because the ionization energy of Li ions is low and the measurement of O elements can also be affected by the Al_2_O_3_ substrate. Fortunately, EDS mappings also can provide qualitative evidence that the distribution of the above-mentioned elements is uniform. Figure 2g–l are the SEM images of S0–S5 at higher magnification. It can be found that the morphology was not influenced by doping with Li ions for the epitaxial films.

### 3.2. Transmittance and UC Luminescence

**Figure 3:**
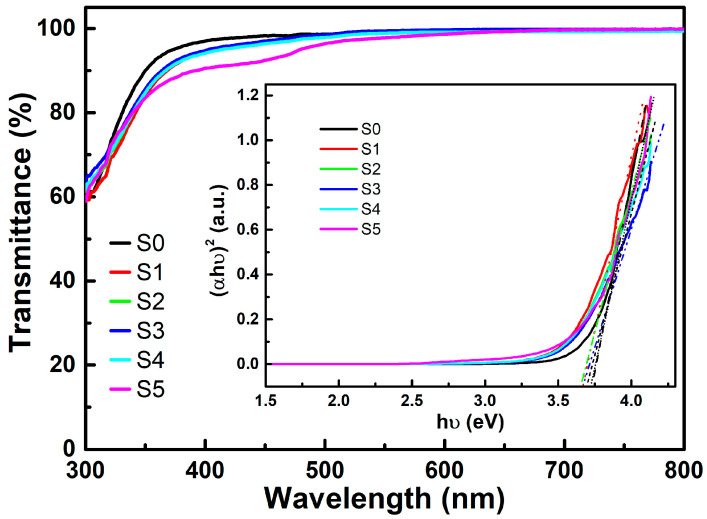
Transmittance spectra of the NiO:Yb,Er thin films after substrate contribution was subtracted. The inset shows the (*αhν*)^2^-*hν* curves for calculating the bandgap.

Figure 3 shows the optical transmittance spectra of pure NiO and doped NiO:Yb,Er thin films. Based on the spectra, the average transmittance of the films can be obtained using the following equation [27]:

(1) $$T_{\mathrm{average}}=\int_{\lambda_{1}}^{\lambda_{n}}T\left(\lambda \right)d\lambda /\left(\lambda_{n}-\lambda_{1}\right)$$

All the obtained samples exhibited high transmittance in the visible spectral range. The pure NiO films have the highest transmittance at 97%. Doping with Li^+^ ions in NiO:Yb,Er films with concentrations below 5% slightly reduced the transmittance to 95%. Sample S5 with the highest Li^+^ concentration still has an average transmittance exceeding 92% in the region between 380 nm and 800 nm. The inset in Figure 3 exhibits the relationship between (*αhν*)^2^ and *hν* for the calculation of the optical bandgap of thin film. The characteristic relation is *αhν* = *B*(*hν* − *E_g_*)^n^, where *hν* is the photon energy, *α* is the related absorption coefficient, *B* is a constant for a certain material, *n* is 1/2 for a material with a direct bandgap, and *E_g_* is the optical bandgap [28]. The value of *α* for a given thickness (*d*) is calculated using the formula *α* = −ln(*T*)/*d* [27], where *T* is the transmittance obtained from Figure 3 and *d* is around 20 nm. The calculated optical bandgap is around 3.7 eV, which corresponds to NiO films in the relevant literature [25]. The large bandgap beyond visible light energy is the reason that the films have a high transmittance in the visible range. These results, together with XRD scans, also prove that the host material of the thin films is NiO and that the doped elements did not have an effect on the basic crystalline structure and visible range transmittance of the host materials.

**Figure 4:**
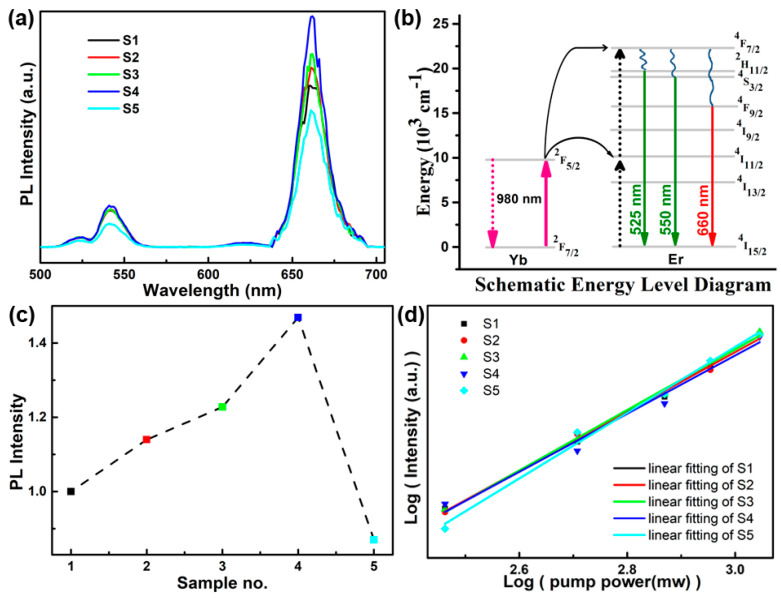
(**a**) PL spectra of NiO:Yb,Er thin films doped with different concentrations of Li^+^ ions. (**b**) Schematic diagram of UC mechanism in the NiO:Yb,Er thin films. (**c**) The integrated intensity of corresponding thin films. (**d**) Pump power dependence of the UC emission intensity at 660 nm of Li^+^-doped NiO:Yb,Er thin films with various doping concentrations.

The PL spectrum of the doped films are shown in Figure 4a under excitation with a 980 nm laser at a laser power of 1000 mW. It can be seen that all the NiO:Yb,Er films with and without Li^+^ doping show clear UC emissions. A two-photon process is considered to be the main UC mechanism in the Yb and Er co-doped materials. Three major emissions were observed at positions around 525 nm, 545 nm, and 660 nm. As shown in Figure 4b, at first, one electron of Yb ion is excited from the ^2^F_7/2_ (Yb) to ^2^F_5/2_ (Yb) level through absorbing a 980 nm photon. Then, the energy can transfer from the ^2^F_5/2_ (Yb) level to ^4^I_11/2_ (Er) level due to their similar energy levels. After that, another 980 nm photon transferred energy from the neighboring Yb ions, exciting the ^4^F_11/2_ (Er) level to the ^4^F_7/2_ (Er) level based on a two-photon process. Subsequently, the high-energy electrons relaxed from the ^4^F_7/2_ (Er) level to the ^2^H_11/2_ (Er), ^4^S_3/2_ (Er), and ^4^F_9/2_ (Er) levels through a non-radiative process. These excited electrons in Er ions returned to the ground state of ^4^I_15/2_ (Er) level, emitting 525 nm (green light), 550 nm (green light), and 660 nm (red light) photons, respectively [29,30]. Furthermore, the influence of Li^+^ ions on UC emission was studied. The red emission intensities of films are summarized in Figure 4c for the comparison of PL intensity. It can be observed that doping with Li^+^ ions in the concentration range between 0% and 5% increases the UC emission intensity. A possible reason is that the doped Li^+^ ions affected the position of Ni^2+^ which reduced the symmetry of the crystal field and then enhanced the UC emission intensity. A similar phenomenon has also be reported in Ce^+^-doped TiO_2_:Yb,Er films and Gd^3+^-doped ErYbGdTiO composite nanophosphor [31,32]. In addition, the relationship between UC intensity and exciting power of the laser is nonlinear, as described in the equation *I* = *P^m^*, where *I* is the UC emission intensity, *P* is the power of the exciting laser, and *m* is the number of photons [31]. As shown in Figure 4d, the slope values of the fitted lines in all samples are approximately 2. This phenomenon indicates that the UC emissions for all Li^+^-doped NiO:Yb,Er films are maintained via a two-photon process.

### 3.3. Conductance Mechanism and Doping-Related Sheet Resistivity

**Figure 5:**
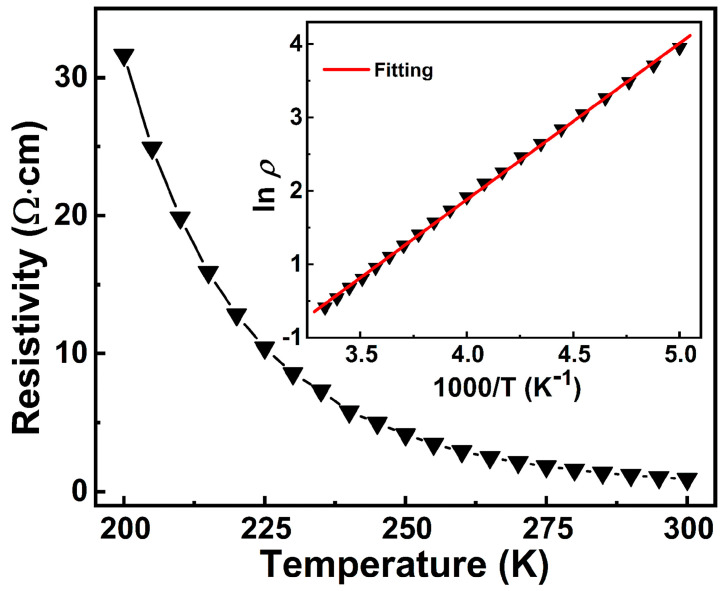
Temperature−dependent resistivity of Li^+^-doped NiO:Yb,Er thin film (S4). The triangles are resistivity of the sample under the corresponding temperature. The inset is ln *ρ* versus 1000/*T* for the film, where the straight line is fit to Equation (2).

The conductance mechanism of the thin films was studied using temperature-dependent Hall effect measurements. Figure 5 shows a typical temperature-dependent resistivity curve of Li^+^-doped NiO:Yb,Er thin film (S4). The resistivity of the film increases when the temperature decreases, indicating a semiconducting behavior. In addition, a positive Hall voltage and Hall coefficient were observed during Hall measurements, which proves that the films are p-type semiconductors. For further analyzing the transport properties, the classic Arrhenius conduction model was used:

(2) $$\rho \left(T\right)=\rho_{0}exp(W/2k_{B}T)$$

where *ρ* is the resistivity, *ρ*_0_ is the proportionality constant, *W* is thermal activation energy, and *k_B_* is the Boltzmann constant [33]. As shown in the inset of Figure 5, the temperature-dependent resistivity curve is well fitted using the Arrhenius conduction model in the measured temperature range from room temperature to 200 K. The value of thermal activation energy is around 360 meV, which was calculated based on the slope of the fitting curve. Compared with the stoichiometric NiO, which have a large bandgap of 3.75 eV, the doped Li^+^ ions with a low activation energy could support acceptor levels and lots of holes in the film, resulting in p-type conducting behavior [34,35].

**Figure 6:**
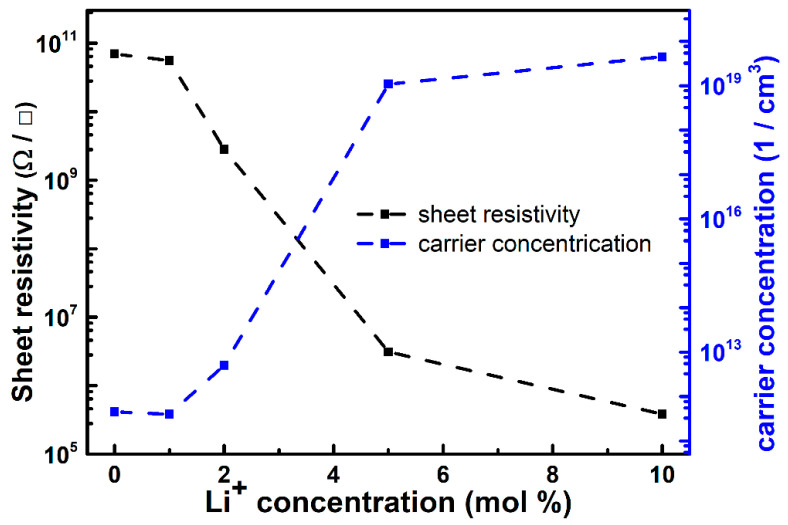
Resistivity and carrier concentration change of Li^+^-doped NiO:Yb,Er thin films at different Li doping concentrations.

We further studied the effect of Li^+^ doping concentration on sheet resistivity and carrier concentration of the thin films, as shown in Figure 6. The related electrical characteristics are also summarized in Table 1 for comparison. Pure NiO (S0) and NiO:Yb,Er (S1) have a similar bandgap and electrical properties, indicating that the role of the doped rare-earth elements is realizing UC emission. The sheet resistivity of Li^+^-doped NiO:Yb,Er films exhibits monotonic decreases, as shown in Figure 6. It also can be observed that a low doping concentration of Li^+^ ions reduced the sheet resistivity of the films. In particular, when doping with 5% Li^+^ ions, the sheet resistivity reduced as large as several orders of magnitude, and the carrier concentration drastically increased. More doping of Li^+^ ions slightly reduced the sheet resistivity, as in Figure 6, but reduced the UC emission properties as discussed in Figure 4.

## 4. Conclusions

In summary, Li^+^-doped NiO:Yb,Er thin films were successfully obtained via pulse laser deposition. All the films have a pure bunsenite NiO phase without any impurity. The average transmittance of all the obtained thin films was higher than 92% in the visible region. Photoluminescence spectra show that all the films have clear red UC luminescence under 980 nm excitation. The UC emission intensity slightly increases with low doping concentrations of Li^+^ and decreases when the doping concentration is above 5%. The temperature-dependent conductivity study reveals a p-type semiconducting behavior of Li^+^-doped NiO:Yb,Er thin films with an activation energy of 360 meV. In addition, it is discovered that Li^+^ ions are an efficient doping element for the improvement of NiO conductance. The sheet resistivity of the film is drastically decreased when doped with 5% Li^+^. This work provides a method to obtain a p-type thin film with excellent transparency, electrical conductivity, and UC luminescence and might be useful for improving the optical and electrical properties of photovoltaics.

## Figures and Tables

**Table 1:** Sample information and electrical characteristics of pure NiO and Li^+^-doped NiO:Yb,Er thin films. The sample no. S0 is pure NiO thin film for reference. The sheet resistivity is expressed as ohms/square, which is abbreviated as Ω/□.

Sample No.	Host Materials	Concentration of Li^+^ (%)	Sheet Resistivity (Ω/□)	Carriers Concentration (1/cm^3^)
S0	NiO	0	7.6 × 10^10^	6.1 × 10^12^
S1	NiO:Yb,Er	0	6.9 × 10^10^	4.5 × 10^11^
S2	NiO:Yb,Er	1%	5.5 × 10^10^	4.1 × 10^11^
S3	NiO:Yb,Er	2%	2.8 × 10^9^	5.1 × 10^12^
S4	NiO:Yb,Er	5%	3.1 × 10^6^	1.1 × 10^19^
S5	NiO:Yb,Er	10%	3.8 × 10^5^	4.5 × 10^19^

## Data Availability

Not applicable.
